# Scene text detection via extremal region based double threshold convolutional network classification

**DOI:** 10.1371/journal.pone.0182227

**Published:** 2017-08-18

**Authors:** Wei Zhu, Jing Lou, Longtao Chen, Qingyuan Xia, Mingwu Ren

**Affiliations:** School of Computer Science and Engineering, Nanjing University of Science and Technology, Nanjing, Jiangsu, China; Beijing University of Technology, CHINA

## Abstract

In this paper, we present a robust text detection approach in natural images which is based on region proposal mechanism. A powerful low-level detector named saliency enhanced-MSER extended from the widely-used MSER is proposed by incorporating saliency detection methods, which ensures a high recall rate. Given a natural image, character candidates are extracted from three channels in a perception-based illumination invariant color space by saliency-enhanced MSER algorithm. A discriminative convolutional neural network (CNN) is jointly trained with multi-level information including pixel-level and character-level information as character candidate classifier. Each image patch is classified as strong text, weak text and non-text by double threshold filtering instead of conventional one-step classification, leveraging confident scores obtained via CNN. To further prune non-text regions, we develop a recursive neighborhood search algorithm to track credible texts from weak text set. Finally, characters are grouped into text lines using heuristic features such as spatial location, size, color, and stroke width. We compare our approach with several state-of-the-art methods, and experiments show that our method achieves competitive performance on public datasets ICDAR 2011 and ICDAR 2013.

## Introduction

Reading text in the wild is significant in a variety of advanced computer vision applications, such as image and video retrieval, scene understanding and visual assistance, since text in images usually conveys valuable information. Hence, detection and recognizing text in scene images has received increasing attention in this community. Though extensively studied in recent years, text detection in unconstrained environments is still quite challenging due to a number of factors, such as high variation in character font, size, color, orientation as well as complicated background and non-uniform illumination.

Previous works for scene text detection based on sliding windows [1–5] and connected component analysis [6–14] have become mainstream in this domain. Sliding windows based methods localize text regions by shifting a multi-scaled classification window. This exhaustive search is computationally inefficient though it achieves high recall rates. Methods based on connected components extract individual characters through connected component analysis followed by grouping and refinement strategy. Additionally, false alarm removing may be performed to remove non-text components. Stroke Width Transform (SWT) [6] and Maximally Stable Extremal Region (MSER) [15] are two representative techniques, particularly methods based on MSER achieved the state-of-the-art performance on ICDAR2013 and ICDAR2015 competitions [16, 17]. However, the MSER algorithms extract massive repeating non-text components which will be constrained by false-removing and refinement rules. These methods are also incapable of detecting characters distorted by noise or background.

More recently, several deep learning based approaches [5, 18–24] have been developed for scene text detection owing to deep model feature representations. These models building on convolutional neural networks (CNN) compute high-level deep features from image patches or proposals for text/non-text classification. These methods are also restricted by region proposal methods and the discriminative power of CNN classifiers.

In this paper, we propose a robust approach which combines the advantages of both MSER and CNN feature representations. Our contributions can be summarized into three points. First, a saliency enhanced-MSER, which is an extension of the well-known MSER algorithm by incorporating saliency detection methods, is proposed as character candidate extractor on three channels of the image to ensure a high recall rate. The second contribution is a novel text filtering pipeline with a deep CNN. In the classification stage, we train a powerful convolutional neural network which incorporates pixel-level and character-level information. The CNN is jointly learned with one main task (i.e., text/non-text classification) and two auxiliary tasks (i.e., text region segmentation and character recognition). With the powerful CNN, we classify the candidates into strong/weak texts and non-texts by applying double threshold filtering. Third, we propose a recursive neighborhood search algorithm to further track texts from strong texts. Finally, we use heuristic rules to construct text lines.

**Fig 1:**
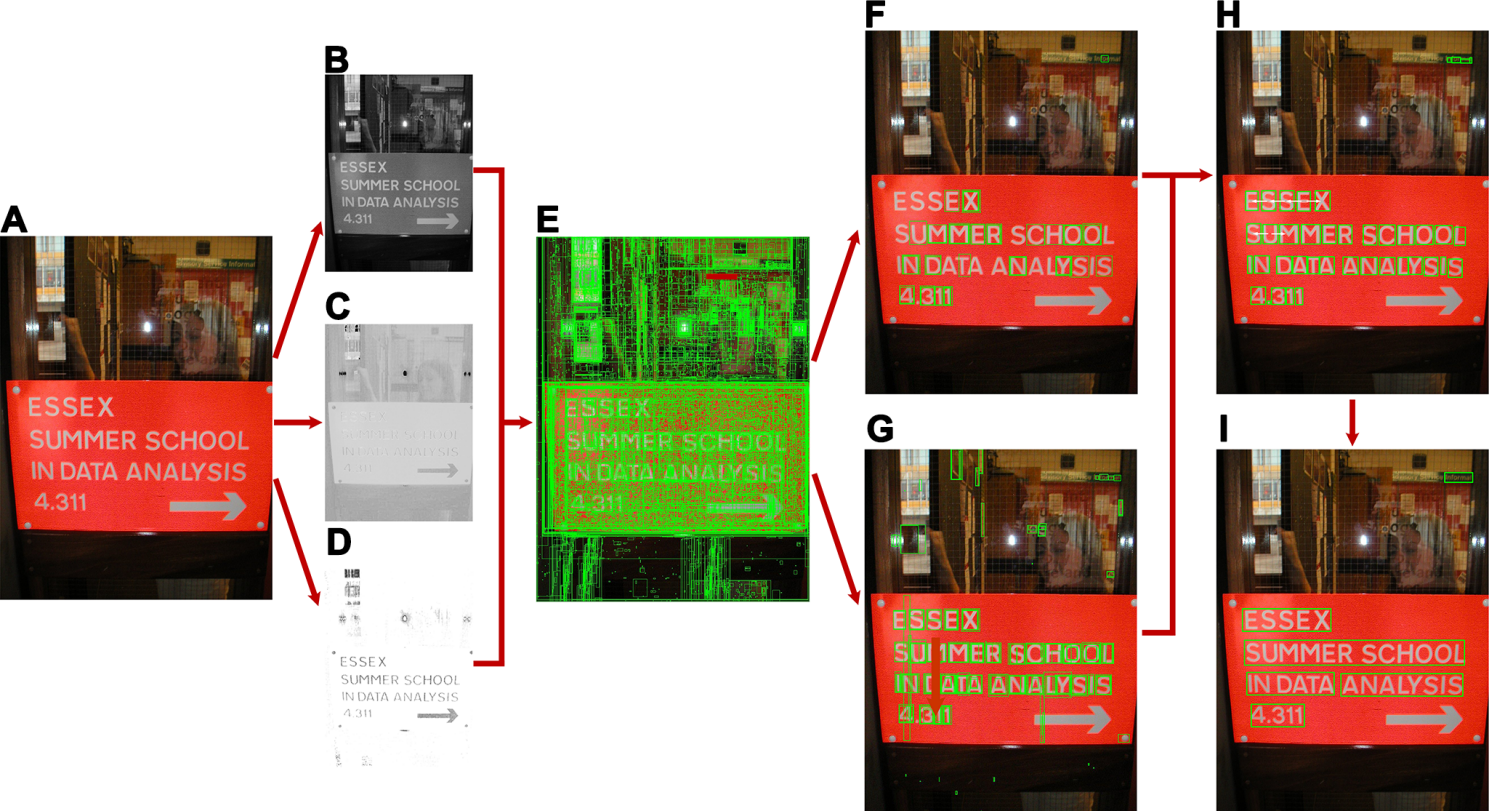
Framework of our proposed algorithm. (**A**) Input image [53]. (**B**) I-channel image in the PII color space. (**C**) H-channel image in the PII color space. (**D**) S-channel image in the PII color space. (**E**) Result of SE-MSER algorithm. (**F**) Strong text after CNN classification. (**G**) Weak text after CNN classification. (**H**) Recursive local search and duplicate removal. (**I**) Final result.

The rest of the paper is organized as follows. In Section *Previous Work*, a brief overview of related studies is given. Section *Methodology* presents the details of the proposed method. Experimental verifications are presented in Section *Experiments and Results*, and finally the paper is concluded in Section *Conclusions*. The pipeline is shown in Fig 1.

## Previous work

Numerous methods have been developed for text localization in real world images in recent years, which can be roughly categorized into two groups: sliding window based methods and connected component based methods. Sliding window based techniques [1, 3, 5, 25] apply multi-scale windows across the image and a character or a word is checked by a classifier. The main limitation is the heavy computational cost resulted by discriminating a huge number of windows.

The connected component based (CC-based) methods [4, 6–15, 19, 26–31] have become increasingly explored in text detection tasks. Stroke Width Transform (SWT) [6] and its variants [4, 10, 11, 13] make use of the property that characters have nearly constant stroke width. These methods are sensitive to noise and blur as they rely on accurate edge detections. The recently most successful methods based on MSER [32] have demonstrated promising performance in the literature. The method presented in [15] detects characters as MSERs followed by a classification process. Neumann and Matas presented a method that considered all extremal regions as character proposals followed by pruning with the exhaustive search strategy. The winning method of ICDAR 2013 [17] in text localization proposed by Yin et al. [29] refines MSER with several pruning techniques and then uses a single-link clustering algorithm to group the characters. The methods in [8, 33, 34] leverage an inclusion relation amongst ERs called ER tree to extract character candidates. For modeling image patches, Baochang Zhang et al. [35] developed multiple Gaussian uncertainty theory and exploited the application in computer vision tasks.

Applying proper features to text plays an important role in the following classification step. In early works, methods in [1, 2] treat text as a special type of texture and make use of its textural properties, such as local intensities, spatial variance, filter responses and wavelet coefficients. Compared with faces and pedestrians, text-lines in natural images have more variations which cannot be well captured by conventional descriptors. Approaches of [7, 10, 13, 27, 30, 36] eliminate non-text components using features based on geometric and appearance properties. Neumann and Matas [7] filtered non-text ERs by cascade filtering using geometric features (e.g. bounding box, perimeter, Euler numbers, horizontal crossings, aspect ratio, compactness, etc.). Yao et al. [10] proposed component level features (e.g., contour shape, edge shape, width variation, density, etc.) to further reject false detections. Huang et al. [13] proposed two novel Text Covariance Descriptors (TCDs) that encode both heuristic properties and statistical characteristics of text strokes. More conventional features and their variants such as LBP, DCT and HOG [28, 33, 34, 37, 38] have been adopted to train classifiers due to their effectiveness. These features are used to train various classifiers such as SVM, random forest and decision trees [7, 28, 39, 40] or construct dictionaries [41–43] for further processing.

Due to the powerful discrimination ability of deep CNN features, various methods based on CNN have been successfully applied to scene text detection recently [5, 18, 20, 21, 23]. Wang et al. [18] employed a traditional CNN model in the sliding window fashion for text detection. In [19, 21], Huang et al. proposed a novel framework which integrated MSER and CNN. The MSER works in the front-end to extract text candidates, while a CNN model is employed to filter out non-text components. This algorithm shows great advantage on performance over conventional methods. Method presented in [5] computes a text saliency map by evaluating the character/background CNN classifier in a sliding window fashion across the image. Gupta et al. [23] developed a Fully-Convolutional Regression Network (FCRN) trained with synthetic images which performs both text detection and bounding box regression. A robust object representation which is a fusion of handcraft features and deep learned features is proposed in [44].

The proposed approach combines the advantages of both text proposal methods and deep CNN models. Despite the success of CC-based methods, we observe that constraints commonly exist in two aspects. First, region proposal techniques are not enough to preserve various true characters, leading to a low recall in practice. Second, text/non-text classifiers are not discriminative enough to reduce the noises in character candidates. Moreover, simply relying on one-step filtering is not robust to detect true texts precisely. Thus, this paper aims to address such limitations.

## Methodology

In this section, we present the details of the proposed algorithm. The full process is separated into three parts: character proposal, text/non-text filtering and text line construction, each of which will be described in details in the next several sections.

### Character candidate extraction

#### Color space conversion

Text is usually perceptually distinct in color from its background, so a color space named perception-based illumination invariant color space which is robust to spectral changes in illumination is used [45]. Let’s assume that $\vec{x}$ is the tristimulus value of sensor represented in *XYZ* coordinates and $F(\vec{x})$ is the 3D color space parameterization. Following [45], the relationship between $\vec{x}$ and $F(\vec{x})$ can be represented by Eq (1) as follows:

$$F(\vec{x})=A(\overset{\wedge }{\ln}(B\vec{x})),$$ (1)

where *A* and *B* are invertible 3×3 matrices and $\overset{\wedge }{\ln}$ denotes component-wise natural logarithm. In [45], the matrices *A* and *B* have been experimentally estimated using databases of similar colors and their values are as follows:

$$A=\left[\begin{array}{ccc} 27.07439 & -22.80783 & -1.806681\\ -5.646736 & -7.722125 & 12.86503\\ -4.163133 & -4.579428 & -4.576049 \end{array}\right]$$ (2)

$$B=\left[\begin{array}{ccc} 0.9465229 & 0.2946927 & -0.1313419\\ -0.117917 & 0.9929960 & 0.007371554\\ 0.0923046 & -0.04645794 & 0.9946464 \end{array}\right]$$ (3)

By transforming the tristimulus values of an image according to Eq (1), one can obtain color descriptors that are approximately invariant to illumination. Therefore, it is intuitive to take advantage of these illumination-invariant color descriptors to extract characters instead of working directly on RGB values. It has been shown in [27] that the PII color space can enhance the robustness of MSER/ER based algorithms.

#### MSER component extraction

**Fig 2:**
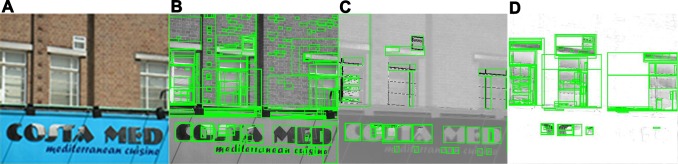
Multi-channel MSER components extraction. (**A**) Original image [53]. (**B**) MSER on gray channel. (**C**) MSER on PII-hue channel. (**D**) MSER on PII-sat channel.

Maximally stable extremal region (MSER) [32, 46] and its variants have been identified as one of the best character region detectors in recent years and demonstrate remarkable performance [19, 29, 31]. However, the assumption that texts usually have distinct contrast to its background and uniform intensity or color may not always hold. MSERs detected as text regions are easily distorted by various factors (e.g., low contrast, low resolution, blurring, etc.), which will lead to numerous false detections. In this step, we focus on retrieving text components as many as possible, so a high MSER margin is used and most ERs are employed. Many recent works [7, 28, 33, 47] have exploited multi-channel techniques to enhance the performance of MSER. An experimental validation in [7] shows that the combination of intensity, hue and saturation channels is found as the best trade-off between short run time and localization performance. In this paper, we extract regions on the grayscale, hue and saturation channel images in the PII color space to ensure the recall rate. Multi-channel MSER detection results can be seen in Fig 2, here we set the MSER threshold to 4 for better display.

#### Saliency-enhanced MSER

Although MSER operator dramatically reduces the number of windows compared with sliding-window methods, some text regions may be missed or distorted resulting in low recall rate in practice. Employing all ERs gets higher recall, the reason we do not apply this is that it suffers from a much larger number of false detections. It is difficult to recover the missed texts in the subsequent progress, thus we need to further improve the recall of the aforementioned MSER method to find a better trade-off between computational cost and detection performance. Towards this, we propose an efficient approach which incorporates cluster-based and histogram-based saliency detection method to enhance region contrast of natural images.

Motivated by cluster-based saliency detection method in [48], we first compute contrast cues from the image. Given an image *I*, we obtain *K* clusters ${\left\{C^{k}\right\}}_{k=1}^{K}$ using K-means algorithm. The contrast cue *w*(*k*) of cluster *C*^*k*^ can be computed using its feature contrast to all other clusters:

$$w(k)=\sum_{i=1,i\neq k}^{K}\left(\frac{n^{i}}{N}{\left\|\mu^{k}-\mu^{i}\right\|}_{2}\right),$$ (4)

where *n*^*i*^ and *N* represent the pixel number of cluster *C*^*i*^ and the whole image, respectively. *u*^*i*^ denotes the cluster center associated with the cluster *C*^*i*^. It is obvious that the larger clusters play more important roles. This approach can strongly enhance the contrast of most dominant or large regions. Unlike [48], we do not compute spatial cues in that texts in images do not strictly satisfy ‘central bias rule’ (i.e., the regions near the image center draw more attention than the other regions). We call the original MSER extraction on cluster-based saliency map as C-MSER for simplification.

We further apply color histogram-based contrast method inspired by [49] to enhance contrast of the small-size regions. Due to the fact that human vision cannot distinguish subtle difference between two similar colors, we reduce color numbers by color quantization which also greatly reduces the computational complexity of color differences computation. Cheng et al. [49] applies uniform quantization which uniformly quantizes each channel of RGB model to 12 different values. However, we employ minimum variance quantization proposed by Heckbert [50] because of the fact that uniform quantization does not take the non-uniform color distribution of a natural image into considerations. Minimum variance quantization constructs a new color map which allocates more entries to colors that appear frequently, and fewer ones to that appear infrequently [51]. Thus, small-size regions assigned with fewer entries in the output color map retain the differentiation and rarity. In this work, we quantize the 24-bit RGB input to 8-bit output with minimum variance quantization which reduces the number of colors to 256.

After quantization, we compute its color histogram by counting the numbers of each color in the RGB color space. Considering that colors in a natural image typically cover only a small portion of the full color space, we further abandon 5 percent of the image pixels whose colors occur less frequently. These pixels are replaced by the closest color in the histogram. While the quantization is performed in the RGB color space, color difference is computed in the L*a*b* color space. The saliency value of color *c*_*i*_ is defined as [49]:

$$S(c_{i})=\sum_{j=1}^{n}f_{j}D(c_{i},c_{j}),$$ (5)

where *D*(*c*_*i*_,*c*_*j*_) is the color distance metric between color *c*_*i*_ and *c*_*j*_ in the L*a*b* space, *n* is the number of colors and *f*_*j*_ is the probability that color *c*_*j*_ occurs. In order to reduce noisy saliency results caused by color quantization, we smooth the saliency value of each color by replacing the weighted average of the saliency values of similar colors. The saliency value of color *c* can be defined [49]:

$$S'(c)=\frac{1}{(m-1)T}\sum_{i=1}^{m}\left(T-D(c,c_{i})\right)S(c_{i}),$$ (6)

where *m* is the number of the nearest colors and here we choose *m* = *n*/4. $T=\sum_{i=1}^{m}D(c,c_{i})$ represents the sum of color difference between color *c* and its nearest colors *c*_*i*_. Through smoothing, similar colors are more likely to be assigned similar saliency values, thus reducing quantization artifacts.

**Fig 3:**
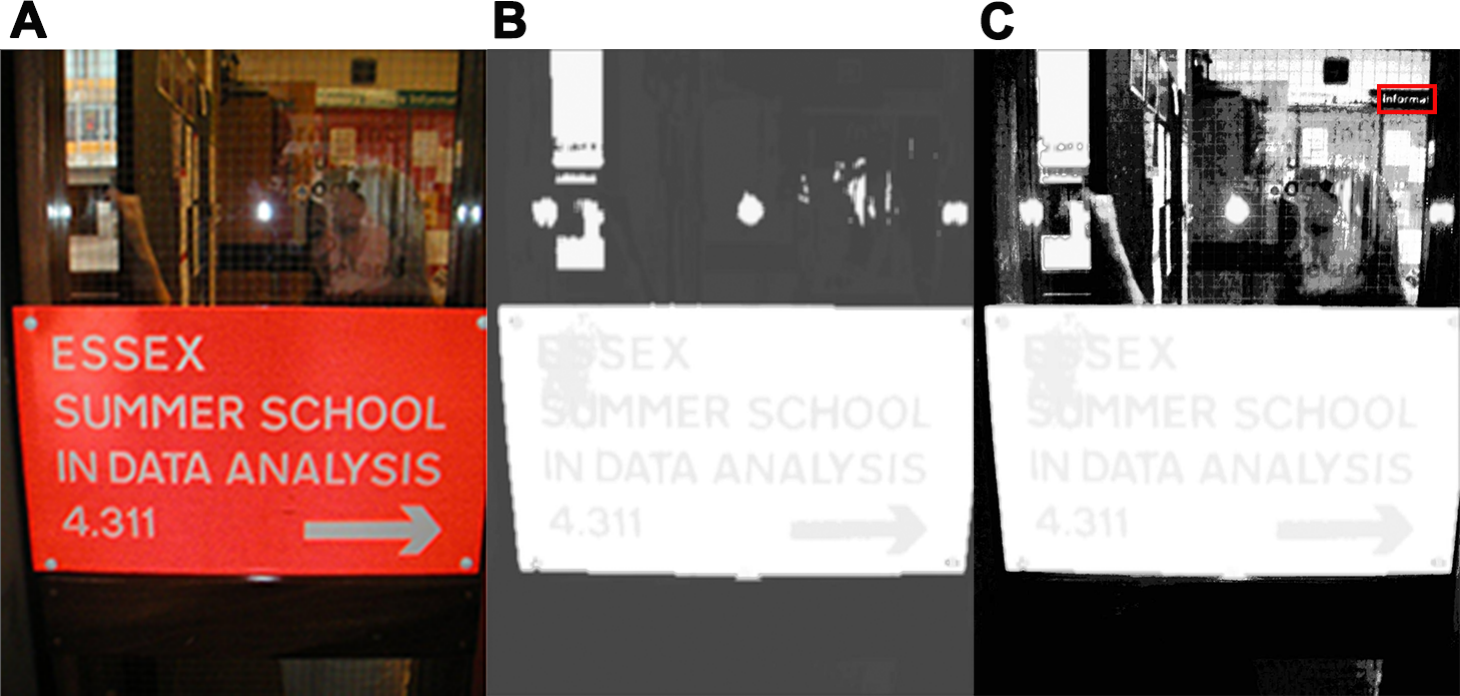
Saliency maps. (A) Original image [53]. (**B**) Cluster-based saliency map. (**C**) Histogram-based saliency map. Note the red rectangle in (**C**) illustrates the missing small characters in (**B**).

The original MSER algorithm is performed on both saliency maps. Finally the results on all channels compose the final character candidates for subsequent processing. We call this saliency- enhanced MSER as SE-MSER for simplification. Results on both saliency maps are illustrated in Fig 3.

### Character candidates filtering

#### Deep text convolutional neural network

To reduce the number of false components detected by MSER, we seek a strong classifier to perform text/non-text classification. Convolutional neural network has been applied to a number of computer vision tasks with remarkable performance achieved in the last few years. Previous works [5, 18, 21] indicate that CNN is capable of learning meaningful high-level feature representations of text components. These approaches either train a character level CNN for scanning an image densely with sliding windows or generate a corresponding heat-map that indicates the probabilities of texts. Due to the fact that humans rely on character information to distinguish text and non-text, we apply a jointly trained deep model presented in [21] which incorporates pixel-level region information and character-level label information.

**Fig 4:**
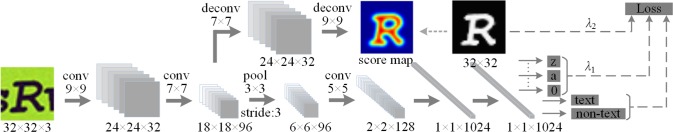
Architecture of text CNN [21].

The structure of our convolutional text network is presented in Fig 4. An input image is first resized to 32×32 and then fed into the network, which is composed of three convolutional layers (with kernel size of 9×9, 7×7, 5×5, respectively) followed by two fully connected layers of size 1024. Each layer is followed by a Rectified Linear Unit (ReLU) as activation function. The second convolutional layer is followed by an additional max pooling layer with kernel 3×3. The last fully connected layer is followed by two softmax layers which perform text/non-text classification and 62-way character classification respectively. Another network branched from the second convolutional layer and composed of two deconvolution layers is the regression model.

The problem is formulated as a multi-task learning (MTL) problem with one main task (i.e., text/non-text classification) and two auxiliary tasks. Given an input image *x*_*i*_, the goal of the MTL problem is to minimize

$$\underset{w^{b},w^{l},w^{r}}{\arg\;\min}L^{B}(y_{i}^{b},f(x_{i};w^{b}))+\lambda_{1}L^{L}(y_{i}^{l},f(x_{i};w^{l}))+\lambda_{2}L^{R}(y_{i}^{r},f(x_{i};w^{r})),$$ (7)

where *f*(∙) is a function of *x*_*i*_ and parameterized by the weight vector w*. The loss function is denoted by ℒ(⋅). λ_*_ denotes the importance coefficient and the regularization terms are omitted for simplification. ${ℒ}^{B}$, ${ℒ}^{L}$ and ${ℒ}^{R}$ indicate text/non-text classification, character label classification and text region regression, respectively. $y_{i}^{b}=\{0,1\}$ (i.e., text/non-text) is the label of the main task, $y_{i}^{l}=\{0\ldots 9,a\ldots z,A\ldots Z\}$ is the label of the character classification task, and $y_{i}^{r}=\{0,1\}$ is 32×32 binary mask of the pixel-level text region. It is reasonable to employ the cross-entropy and least square as loss functions for classification tasks and regression task, respectively.

The training process is identical to [21]. After jointly training the two auxiliary tasks (i.e., text region regression and character recognition), we adopt “task-wise early stopping” method [52] to early stop the region task before the main task starts. The intuition is that low-level task will harm the main task after it reaches its peak performance as training proceeds. The character recognition task continues with training of the main task until the model is finally optimized.

#### Double threshold classification

The text CNN is adopted to filter non-text candidates among all the components detected by MSER. Inspired by previous work [33], the surviving character candidates are classified into three classes: strong text, weak text and non-text. In [33], Cho et al. applies a structure of two blocks of cascaded Adaboost classifiers, which is replaced by the more powerful text CNN, to filter the MSER candidates.

**Fig 5:**
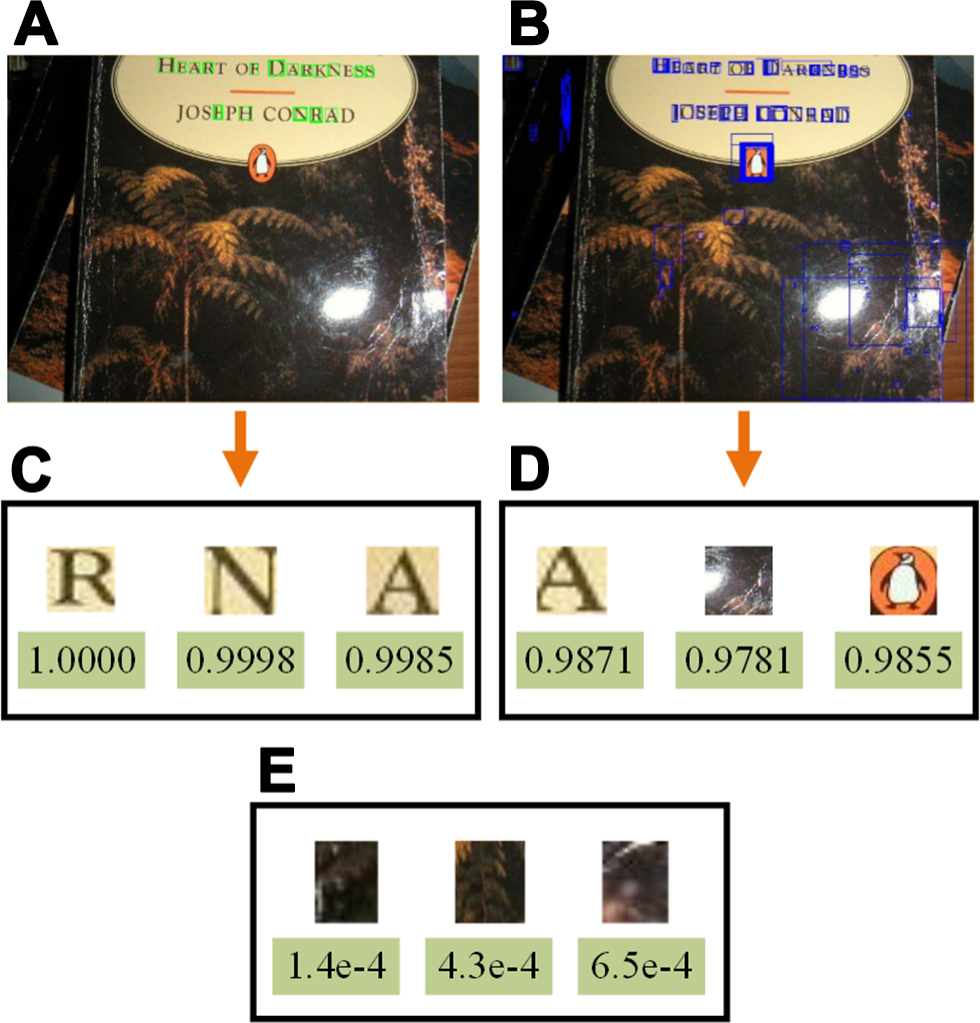
Examples of text candidate classification. (A) Detected strong texts in green boxes. (B) Detected weak texts in blue boxes. (C) Strong text examples with confidence scores. (D) Weak text examples with confidence scores. (E) Non-text examples with confidence scores. The original image is from ICDAR 2011 dataset.

**Fig 6:**
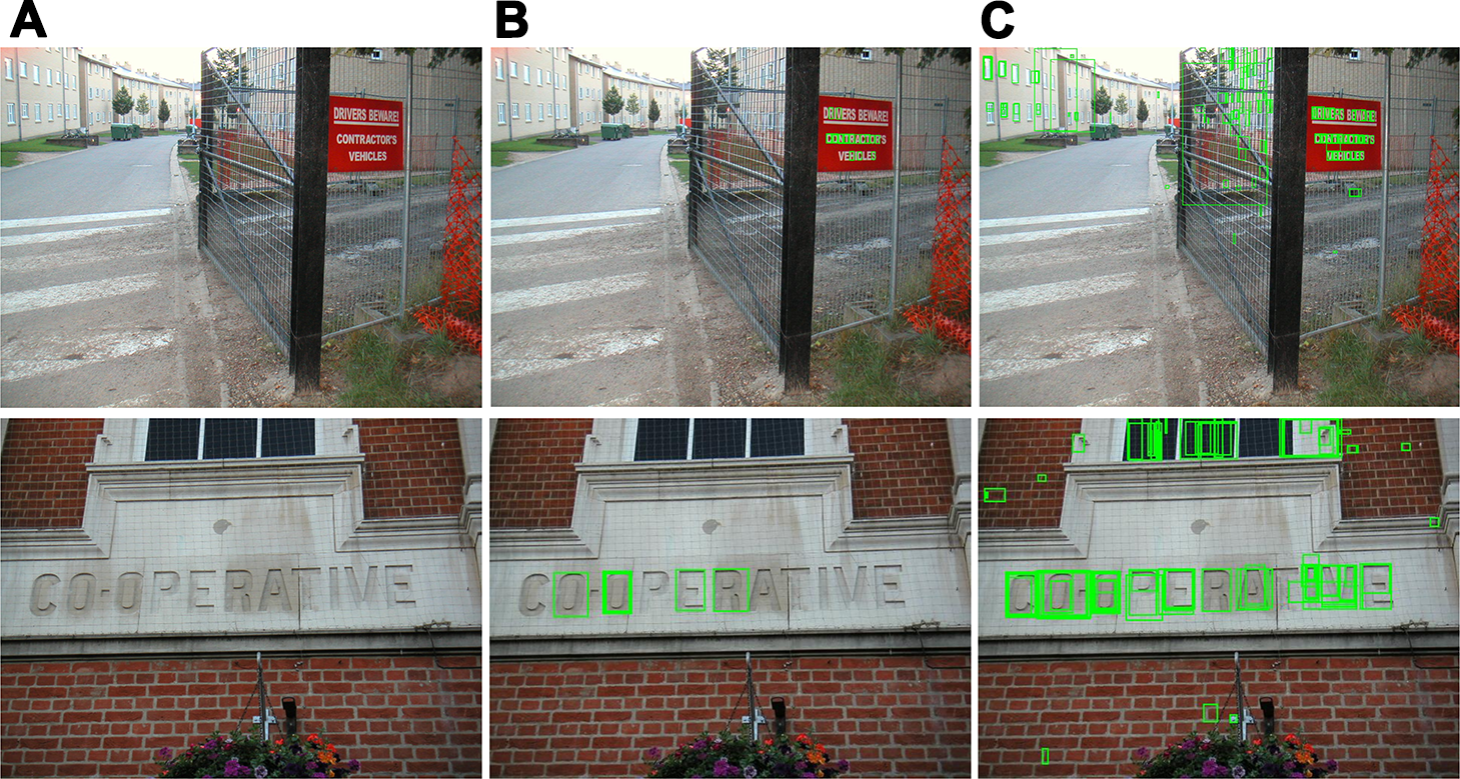
Classification results by double threshold filtering. (**A**) Original images [53]. (**B**) Strong texts. (**C**) Weak texts.

The cropped images of all candidates go through the CNN and the CNN produces a confident score for each of them. It is essential to filter out regions with a low confidence score and preserve those with a high score. This is accomplished by selecting high and low threshold values as follows:

$$c\in \left\{ \begin{array}{l} {ℛ}^{S},s_{c}>T_{h}\\ {ℛ}^{W},T_{l}<s_{c}<T_{h}\\ {ℛ}^{N},s_{c}<T_{l} \end{array}\right.,$$ (8)

where ${ℛ}^{S}$, ${ℛ}^{W}$, ${ℛ}^{N}$ indicate strong text, weak text and non-text, respectively. *s*_*c*_ is the confidence score, and *T*_*h*_, *T*_*l*_ represent the high and low thresholds. Through this, the remaining regions can be separated as strong texts and weak texts, whereas the non-texts are removed from the candidates (see Fig 5). The double thresholds are determined by validation on the training set which satisfy precision of 99% and 90%, and here we set them to 0.995 and 0.978 respectively. Fig 6 shows the classification results with the double threshold.

### Text grouping and refinement

#### Recursive neighborhood search

After candidates labeling, most of the non-text areas are removed while the text components are well preserved. However, some of the weak texts cannot be correctly classified due to low resolution or severe distortion. As nearby text areas share similar properties in the same word or text line, we propose a Recursive Neighborhood Searching (RNS) strategy to investigate positive components from the weak set. Details of the procedure are outlined in Algorithm 1.

**Algorithm 1** Recursive Neighborhood Search

**Input:** Strong text set ${ℛ}^{S}$, weak text set ${ℛ}^{W}$.

**Output:** Positive text set ${ℛ}^{T}$.

1: Initial ${ℛ}^{S}\leftarrow {ℛ}^{S}$;

2: **for each** $c\in {ℛ}^{S}$ from left to right **do**

3:  **for each** $l\in {ℛ}^{W}$ **do**

4:    **if** *Dist*_*cen*_(*c*,*l*) ≤ *T*_1_ × max(*w*_*c*_,*w*_*l*_)

            &*abs*(*w*_*c*_,*w*_*l*_) < min(*w*_*c*_,*w*_*l*_)

            &*abs*(*h*_*c*_,*h*_*l*_) < min(*h*_*c*_,*h*_*l*_)

            &*Dist*_*color*_(*c*,*l*) ≤ *T*_2_

            &1/*T*_3_ < *sw*_*c*_/*sw*_*l*_ < *T*_3_

5:                ${ℛ}^{T}\leftarrow l$;

6:                remove *l* from ${ℛ}^{W}$;

7:   **endif**

8:  **endfor**

9:    **if** ${ℛ}^{W}=\emptyset$

10:            **break**;

11:          **endif**

12: **endfor**

Tracking texts by similar heuristic features.

Here *Dist*_*cen*_(*c*,*l*) denotes normalized *L*2 distance between *c* and *l*, and *Dist*_*color*_(*c*,*l*) stands for color difference. *w*, *h* and *sw* refer to width, height and mean stroke width respectively. The parameter *T*_1_ is set to 2, the threshold *T*_2_ for color difference is set to 25, and *T*_3_ is set to 1.5. Fig 1H shows the tracked texts by RNS.

In our experiments, there exist some inner boxes or outer boxes corresponding to the same text (e.g., partial detected and out of range), which may be a threat to the following text grouping procedure. To address the problem, we apply a robust method to solve this problem. In the first stage, we perform non-maximum suppression based on confident scores among overlapped regions and select the region with the highest score. In the second stage, we merge the region with the remaining regions if their intersection-over-union measure is greater than 0.5. The merging step is processed in descending order of confident scores.

#### Text Line Construction

Given the results of Recursive Neighborhood Searching, the text line construction is straightforward. Similar to previous work [9, 19, 33], we only use some commonly used heuristic rules to group the text candidates into lines. As our searching algorithm has extracted credible characters, the grouping procedure can be performed efficiently based on the same similarity measures (i.e., spatial location, size, color and aspect ratio). Candidates which satisfy the property similarities are grouped sequentially into the same word to construct the final text lines. Minimum bounding boxes that enclose grouped texts are our final results, as shown in Fig 1I.

## Experiments and results

### Evaluation method and datasets

We evaluated the proposed method on two widely cited datasets for benchmarking scene text detection: ICDAR 2011 RRC dataset [53] (the dataset is downloaded from http://www.cvc.uab.es/icdar2011competition), and ICDAR 2013 RRC dataset [17] (the dataset is downloaded from http://rrc.cvc.uab.es). The images of “Challenge 2: Reading Text in Scene Images” are used. The real world images consist of a range of scenes with texts and word-level ground truth is provided. We do not evaluate on more recent ICDAR 2015 dataset [16] as it is almost identical to the 2013 dataset.

We evaluate our algorithm from two aspects: character-level and word-level. For character-level recall evaluation, PASCAL VOC style protocol (i.e., a correct detection is determined if the intersection-over-union between a detected region and ground truth region is over 0.5) is used. For word-level evaluation, we adopt the protocol proposed by Wolf et al. [54]. In this criterion, the matching cases are classified as one-to-one, one-to-many and many-to-many.

### Evaluation of the Saliency-enhanced MSER

**Fig 7:**
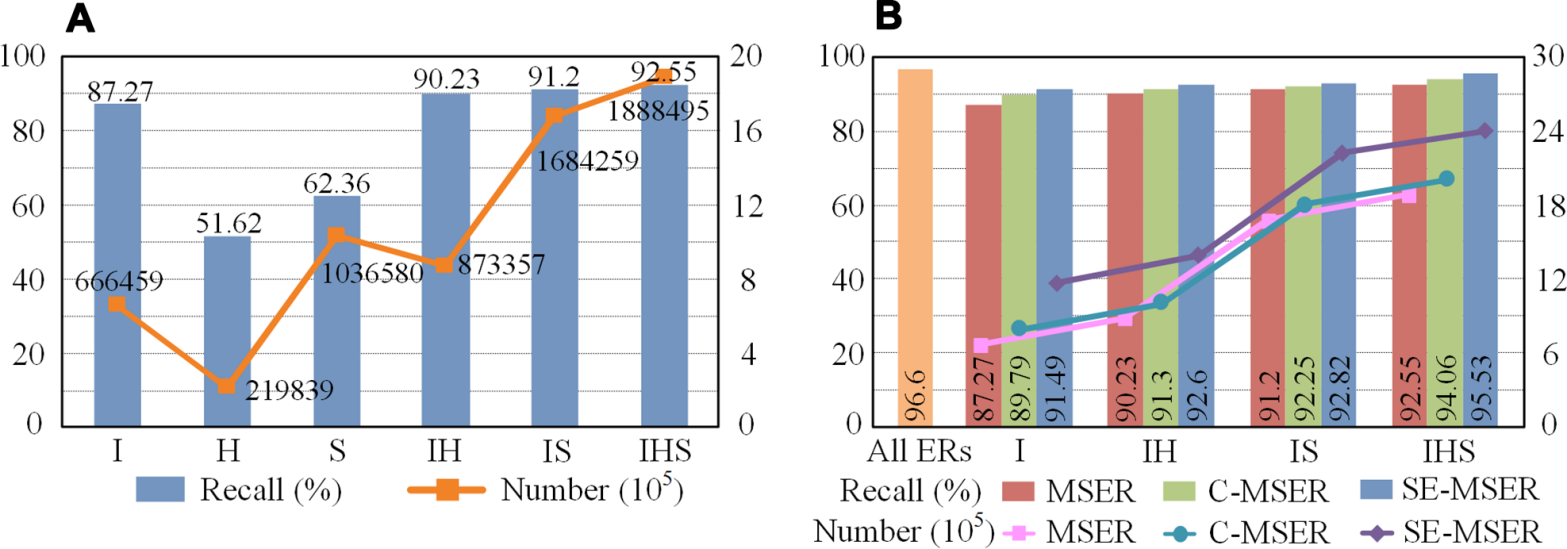
Evaluation of character-level recall on the ICDAR 2011 test set. (**A**) Original MSER on multiple channels. (**B**) Multiple MSERs comparison.

We perform a quantitative analysis of character-level recall on the ICDAR 2011 dataset. The total number of images and characters in the test set are 255 and 6309, respectively. The character-level ground truth data is obtained from Cho H [33]. The character extraction is performed by original MSER algorithm on each channel and combined channels, and the number of candidates is counted additionally (see Fig 7A). The MSER threshold here is set to 1 to ensure a better recall. The result shows that the best recall rate is achieved on the combination of intensity (I), hue (H) and saturation (S) channels, and obviously the intensity channel plays the leading role.

To evaluate the effectiveness of our saliency-enhanced MSER, we compare the recall using original MSER (MSER), cluster-based enhanced MSER (C-MSER) and saliency-enhance MSER (SE-MSER), respectively (see Fig 7B). The orange column indicates that 96.6% characters can be detected if all ERs are extracted, the total number of which is 6051331. Results without intensity channel are not shown for simplification. It is worth mentioning that our saliency-enhanced MSER achieves a comparable recall rate to all ERs with a much smaller number of candidates.

Table 1 shows the comparison results with other character candidate extraction methods. Note that the evaluation method in the first four rows (i.e., a character is detected if the bounding box matches over 90% of the ground truth), which is slightly loose compared with the VOC protocol, is not the same as the rest. The evaluation method mentioned in [7, 29] requires that over 90% of the detected area is matched by a ground truth box for the detection to be considered correct. Therefore, a small detected region contained in a large ground truth box will be considered positive. This problem can be solved by the VOC evaluation method which is based on intersection-over-union. It can be seen that our method using three channels achieves the highest recall rate. Note that we do not discuss the numbers here in that our aim is to extract as many true characters as we can for further processing.

**Table 1:** Character-level recall rate on the ICDAR 2011 test set.

Algorithm	Recall (%)
Neumann and Matas [7] (grayscale)	85.6
Neumann and Matas [7] (four channels)	93.7
Yin et al. [29] (grayscale)	90.2
Yin et al. [29](three channels)	95.2
Sung et al. [34] (three channels)	87.7
Sung et al. [34] (initial ERs)	89.6
Cho H et al. [33]	95.1
Our proposed method	**95.53**

### Experiments on ICDAR2011 and ICDAR 2013

We evaluate our method on the ICDAR 2011 dataset for word-level experiments. Table 2 illustrates the comparison results with some recently published methods. The proposed method achieves 0.836 in F-measure, outperforming other methods. Compared to the closest competitors [19, 33], the precision of our algorithm (0.875) is much higher than both of theirs (0.76 and 0.71), which owes to the high accuracy of detected characters by the high threshold filtering.

**Table 2:** Experimental results on the ICDAR 2011 dataset.

Method	Recall	Precision	F-score
Yi et al. [11]	0.581	0.672	0.623
Epshtein et al. [6]	0.60	0.73	0.66
Kim et al. [47]	0.625	0.830	0.713
Shi et al. [9]	0.631	0.833	0.718
Yao et al. [56]	0.827	0.652	0.730
Neumann and Matas [8]	0.675	0.854	0.754
Yin et al. [29]	0.683	0.863	0.762
Huang et al. [19]	**0.88**	0.71	0.78
Zhang et al. [25]	0.84	0.76	0.80
Proposed	0.801	**0.875**	**0.836**

The performances of the proposed algorithm as well as other methods on the ICDAR 2013 are depicted in Table 3. The proposed method obtains 0.792, 0.884 and 0.835 in recall, precision and F-measure, respectively. The recall and F-measure still outperforms other methods. This confirms the effectiveness of our algorithm, especially its robustness in handling various scenarios.

**Table 3:** Experimental results on the ICDAR 2013 dataset.

Method	Recall	Precision	F-score
Neumann and Matas [8]	0.648	0.875	0.745
USTB TexStar [17]	0.664	0.885	0.759
Neumann and Matas [31]	0.724	0.818	0.771
Zhang et al. [25]	0.74	0.88	0.80
Sung et al. [34]	0.742	0.887	0.808
He et al. [21]	0.723	**0.923**	0.817
Cho et al. [33]	0.785	0.863	0.822
Zhang et al. [57]	0.78	0.88	0.83
Proposed	**0.792**	0.884	**0.835**

**Fig 8:**
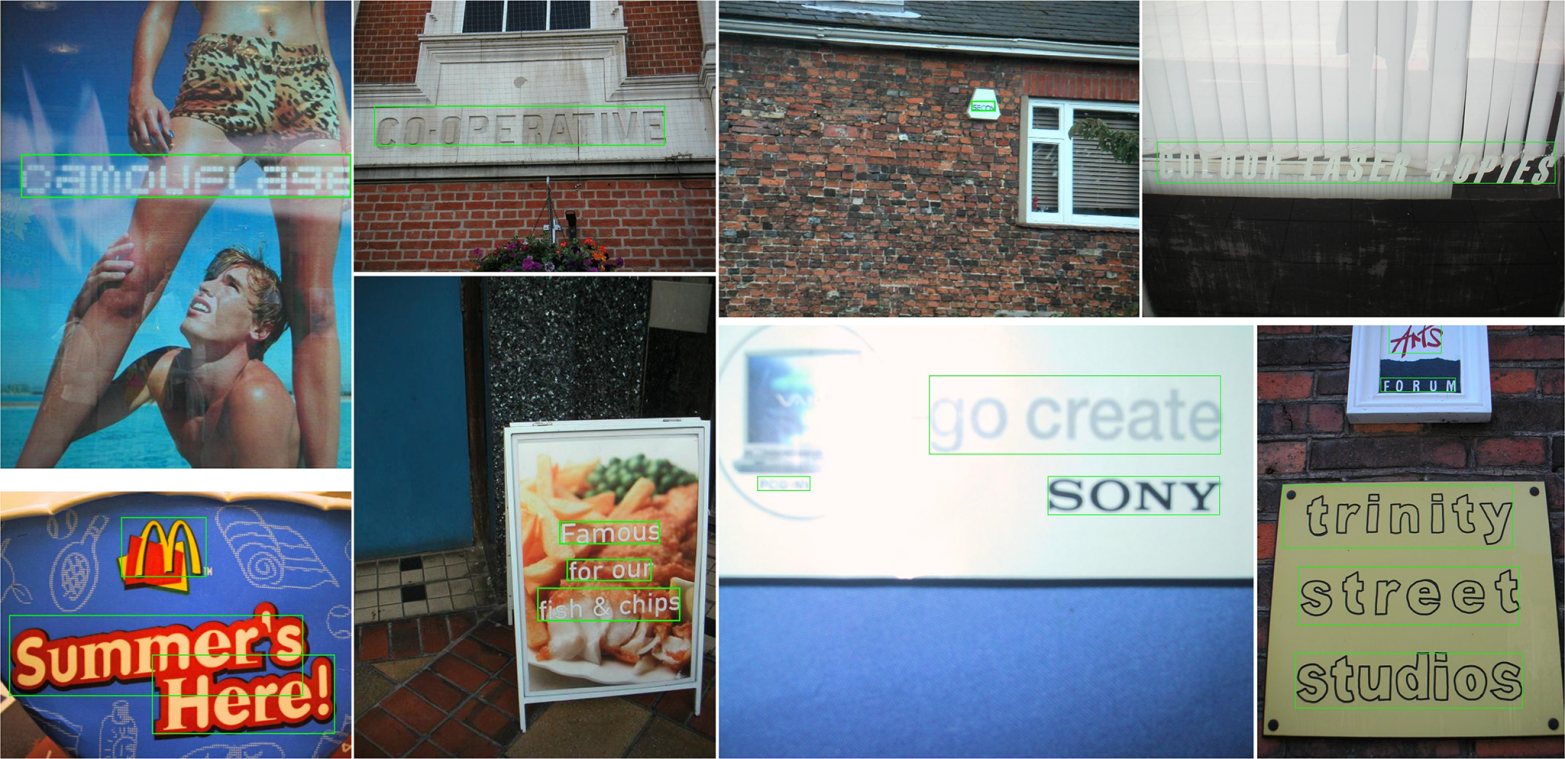
Detection examples of the proposed method on the ICDAR 2011 dataset [53].

**Fig 9:**
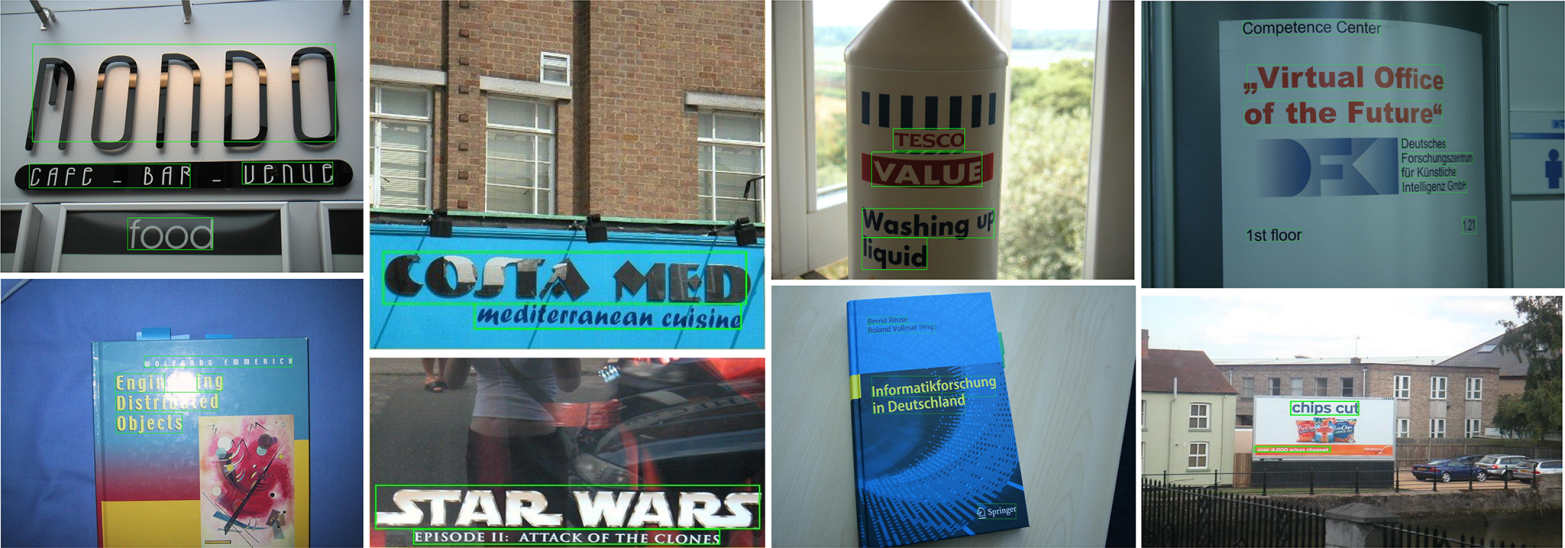
Detection examples of the proposed method on the ICDAR 2013 dataset [17].

Besides the quantitative experimental results, several typical detection examples of the proposed method are shown in Fig 8 and Fig 9. As can been seen, our proposed algorithm works fairly well under various challenging cases (e.g., dot matrix fonts, low resolution and non-uniform illumination), which may not been handled perfectly using conventional methods.

### Limitation of the proposed method

**Fig 10:**
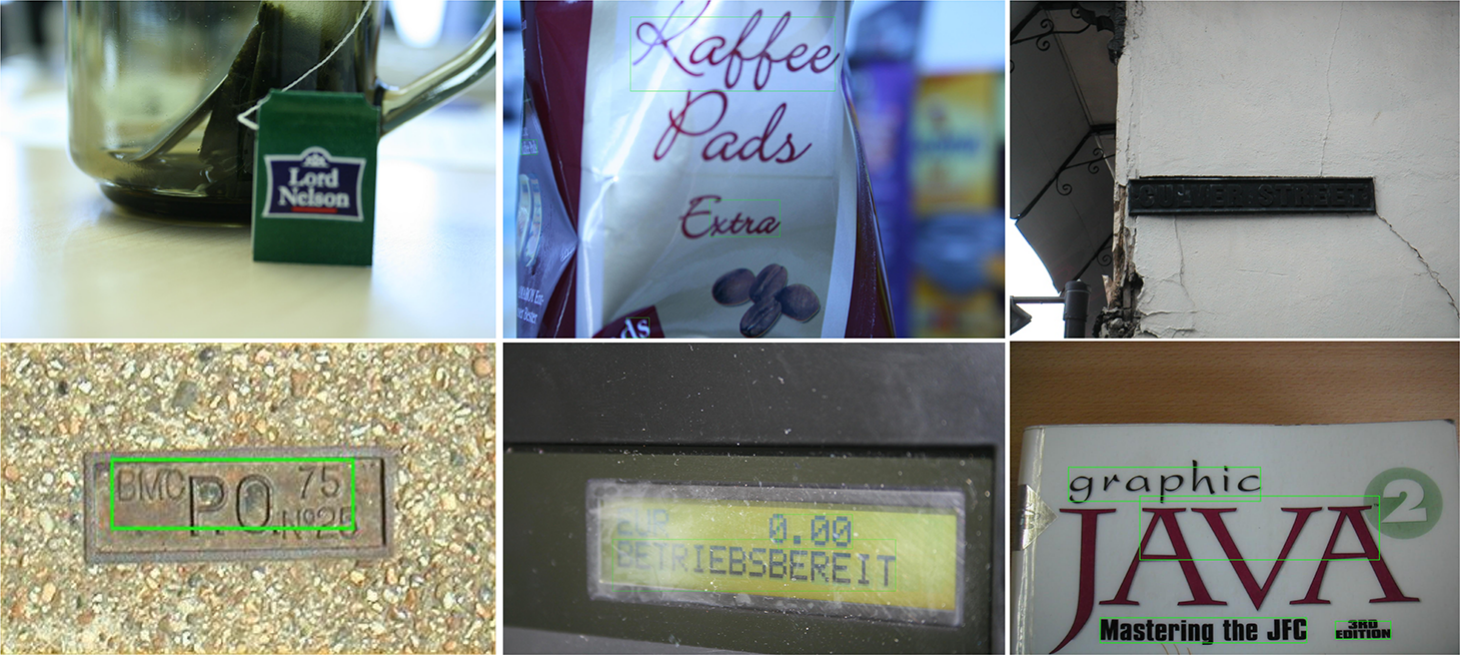
Unsuccessful samples on the ICDAR dataset [17, 53].

The proposed method is capable of dealing with several challenging cases and achieves excellent performance on standard benchmarks. However, there is still a great gap between our result and the perfect performance. Several failed examples are illustrated in Fig 10. As can be seen, false positive and missing characters may appear in certain situations, such as extremely low contrast, overexposure, special alignment, and blurring or tremendous gap between characters. Some cases have extremely ambiguous text information and are even hard for human to distinguish.

Another limitation is the speed of the proposed algorithm. We implemented our algorithm in MATLAB and the CNN was trained in Caffe [55] framework. Our system was benchmarked on a 3.3GHz, 8 cores PC with a Quadro K4000 GPU, running Linux 64bit version. The average running time was evaluated on the ICDAR 2011. The original MSER implemented in MATLAB takes about 980ms and our SE-MSER takes about 3.7s per image. This can be accelerated significantly using C++ with more engineering optimization. The Text-CNN takes about 1.5s per image and the convolutional forward passes can be very fast running powerful GPU devices. The average time of processing one image is about 5.8s, which is slightly faster than the result (7.3s) reported in [20]. However, this speed is still far from the requirement of real-time applications. More efforts need to be made on speeding up the text proposal method.

## Conclusions

In this paper, we have introduced a novel algorithm for text detection in natural images. On the basis of previous successful methods, we extend the advantages of MSER and deep convolutional neural network. Moreover, a double threshold filtering strategy is adopted instead of conventional one-step classification to better capture true texts and remove non-text components. The experiments on the latest ICDAR datasets demonstrate that the proposed algorithm outperforms other competing methods in the literature. In the future work, we will investigate better strategies to reduce the number of proposals efficiently, thus satisfying the requirement to real-time applications.
